# PVP-H_2_O_2_ Complex as a New Stressor for the Accelerated Oxidation Study of Pharmaceutical Solids

**DOI:** 10.3390/pharmaceutics11090457

**Published:** 2019-09-03

**Authors:** Dattatray Modhave, Brenda Barrios, Amrit Paudel

**Affiliations:** 1 Research Center Pharmaceutical Engineering GmbH (RCPE), 8010 Graz, Austria; 2 Institute of Process and Particle Engineering, Graz University of Technology, 8010 Graz, Austria

**Keywords:** PVP-H_2_O_2_, stress study, oxidative stability, reactive excipient impurities, degradation product

## Abstract

Reactive impurities, such as hydrogen peroxide in excipients, raise a great concern over the chemical stability of pharmaceutical products. Traditional screening methods of spiking impurities into solid drug-excipient mixtures oversimplify the micro-environment and the physical state of such impurities in real dosage form. This can lead to an inaccurate prediction of the long-term product stability. This study presents the feasibility of using a polyvinylpyrrolidone-hydrogen peroxide complex (PVP-H_2_O_2_) as an oxidative agent for the solid state forced degradation of a selected drug, vortioxetine HBr. The PVP-H_2_O_2_ complex was prepared and characterized using FT-IR spectroscopy. The tablet compacts were made using a mixture of solid PVP-H_2_O_2_ complex and crystalline vortioxetine HBr powder. The compacts were exposed to 40 °C/75% RH condition in open and closed states for different time intervals. The extent and the type of drug degradation were analysed using LC and LC-MS. The extent of degradation was higher in the samples stored at the open state as compared to the close state. The solution state forced oxidation was conducted to verify the peroxide induced degradation reactions. The results evidence the utility of the proposed solid-state stressor and the method for screening the sensitivity of drugs to the excipient reactive impurities involving peroxides in solid-state.

## 1. Introduction

Pharmaceutical excipients are known to contain trace impurities, such as reactive oxygen species (as hydrogen peroxides and/or organic hydro peroxides) and formaldehyde, often referred to as reactive impurities [1,2]. These reactive species in excipients can lead to the degradation of drug in the drug products. The lot-to-lot variation of reactive impurities content among excipients is a well-recognized challenge to formulation scientists. Small organic molecules and biomolecules including therapeutic proteins are susceptible for such reactive species [3]. Starting from the synthesis, the formulation processing, drying, sterilization, packaging, and storage to the consumption, drug molecules are susceptible to the risk of oxidation. In the industry, the risk of oxidative drug degradation is identified by conducting the forced degradation studies in solution using reagents such as 2,2-azobisisobutyronitrile (AIBN), 4,4-azobis-4-cyanovaleric acid (ACVA), 2,2′-azobis(2-amidinopropane) dihydrochloride (AAPH), and liquid hydrogen peroxide (H_2_O_2_) stressors [4,5]. The validity of such approaches for the accurate prediction of instability in solid state products is often limited [6,7]. The reactive excipient impurities mediated oxidative degradation in a solid-state is challenging to investigate and rarely reported. Some oxidative methods have been reported for the stress studies [8,9], which barely represent the actual solid-state environment in the presence of the relevant excipient.

The present study deals with the preparation of a hydrogen bonded polyvinylpyrrolidone-hydrogen peroxide (PVP-H_2_O_2_) molecular complex and its use as a solid-state reagent for the oxidative study of crystalline vortioxetine HBr. The polymeric complex of peroxide was prepared by modifying the method reported in the literature [10] and characterized by infrared spectroscopy. Vortioxetine HBr was selected, owing to its selectivity, to undergo peroxidation as the main degradation reaction [11]. The powder compacts containing the physical mixture of the drug and the PVP-H_2_O_2_ complex were subjected to accelerated storage at 40 °C/75% RH, and the degradation product was subsequently quantified using LC-PDA/MS. The common oxidative stressors are listed in Table 1 with their scope and shortcomings [12].

## 2. Materials and Equipment

### 2.1. Chemicals and Reagents

Vortioxetine hydrobromide (VOR) was purchased from Shenzhen Nexconn Pharmatechs Ltd. (Shenzhen, China) The reagents, including hydrogen peroxide solution (30% *w*/*w*) and hydrogen peroxide-urea adduct solid powder, were purchased from Sigma-Aldrich (St. Louis, MO, USA) and stored at refrigerated conditions at 2–8 °C. Polyvinylpyrrolidone (PVP) K-30 powder was obtained from BASF SE (Ludwigshafen, Germany). All other chemicals used in the study were of analytical reagent grades.

### 2.2. Equipment

Particle size distribution analysis was performed for VOR powder using QICPIC analysis (400 fps) equipped with RODOS dry dispersion unit (Sympatec GmbH, Clausthal-Zellerfeld, Germany). Brunauer, Emmett, and Teller (BET) surface area measurement was performed on the drug-solid stressor compacts using nitrogen physisorption (TRISTAR II, Micromeritics, Norcross, GA, USA). The degradation samples were analyzed using Acquity ultra-high-pressure liquid chromatography (UHPLC) equipped with photodiode array ultraviolet and single quadrupole mass detector (Waters, Milford, MA, USA). The simultaneous DSC-TGA from NETZSCH STA 449 C (NETZSCH, Selb, Germany) was used for the determination of moisture content. The infrared spectral studies were performed using Vertex 70 with ATR sampling unit (Bruker Optik GmbH, Ettlingen, Germany).

## 3. Experimental and Methods

### 3.1. Oxidative Forced Degradation Study in Solution and Solid State

Liquid hydrogen peroxide (30% H_2_O_2_) was used to generate oxidative degradation species in solution. Approximately, 10 mg of VOR was added to 10 mL of reagent solution and kept at room temperature for 3 h. The treated solution was diluted two times using dilution solvent and analyzed by UHPLC. The forced degradation study in solid state was assessed using urea-hydrogen peroxide (UHP) powder as a stressor. Zhu et al. reported the generation of peroxide vapor using UHP to evaluate oxidative stability of pharmaceuticals [9]. In contrast, the intention of using UHP directly as powder blend with the API in our study was to generate a higher amount of degradate through a high peroxide activity (contains 15–17% active oxygen basis). A higher amount of the expected degradation using such reagents make spectroscopic detection easier for detection, for example, by FTIR spectroscopy. A mixture containing 50 mg of drug and 50 mg of UHP powder were compressed in the form of a compact disc. The drug-stressor (1:1) compacts were exposed to 40 °C/75% RH for 24 h. Afterwards, they were fully dissolved in the diluent (100 mL) and analyzed by UHPLC.

### 3.2. Establishment of the Analytical LC Method 

The oxidative stress samples from the solution state forced degradation were used to develop the stability indicated LC method. A reversed phase chromatographic method was used to separate the drug and the degradation products. The LC parameters include C18 column (HSS T3 1.8 μm, 2.1 × 100 mm, Waters, Milford, MA, USA) for elution and 0.05% trifluoroacetic acid and acetonitrile were used as mobile phase solvents A and B, respectively. The solvent ratios were changed appropriately in a gradient mode for a period of 11 min (*T*_min_/A:B; *T*_0.00_/80:20; *T*_1.00_/80:20; *T*_5.00_/30:70; *T*_10.00_/30:70; *T*_11.00_/80:20) with 5 min of the end equilibration. During the run, the flow rate was kept constant at 0.35 mL min^−1^ and the injection volume was set at 1 μL. The column oven was set at 30 °C and the autosampler was at 20 °C. The UV detection was carried out at a wavelength of 242 nm. A mixture of acetonitrile and water in the ratio of 50:50 *v*/*v* was used as the sample dilution solvent. The final active concentration injected was 500 µg/mL. The percentage of degradation reported was based on the area under the curve (AUC) of the chromatographic peaks (relative response corresponding to peaks of degradate and VOR). The LC method was selective and linear over the concentration range of 0.6 µg/mL to 600 µg/mL.

### 3.3. Liquid Chromatography-Mass Spectrometry (LC-MS) Method

The LC-MS studies were performed on VOR stress samples solution using electrospray ionization (ESI) in a positive mode to obtain the nominal mass values. The capillary and cone voltage were kept at 3 kV and 35 V, respectively. The desolvation temperature, ion source temperature and desolvation gas flow rate (nitrogen) were 400 °C, 150 °C and 600 L/h, respectively. Since the detailed structural elucidation of the degradate was out of the scope of the present study, the obtained line spectra corresponding to the molecular ion peak for VOR and the degradate were compared with the literature reports for interpretation [11].

### 3.4. Preparation of Solid State Stress PVP-H_2_O_2_ Complex (PHP Complex)

The PVP K-30 powder and the 30% H_2_O_2_ solution were used as starting material to obtain a solid complex reagent. The complex is hereafter denoted as the PHP (PVP hydrogen peroxide) complex. For the preparation, 12 gm of PVP K30 powder was added to 18 mL precooled (using ice bath) 30% *w*/*w* H_2_O_2_ solution in a glass beaker. The solution was stirred continuously at 250 rpm for 1 h. The resultant solution was transferred to another glass beaker containing Teflon film and then kept on a bench for 15 h at 25 °C. Further, drying of the sample was carried out by keeping it in desiccator (vacuum tightened) for 35 d at 40 °C. The solid powder obtained after drying was crushed using mortar and pestle assisted with liquid nitrogen. The obtained PHP solid powder was stored at 2–8 °C. The reproducibility of the preparation was ensured by repeating the experimental procedure three times.

### 3.5. Measurement of pH of the PHP Complex

Approximately 100 mg PHP was dissolved in 1 mL of distilled water. The container was exposed for a couple of minutes to the ultrasonic to completely dissolve them. The pH electrode was washed with distilled water before and between each measurement.

### 3.6. ATR-FTIR Spectral Analysis

Fourier-transform infrared (FTIR) spectroscopy of the solid PHP was performed using attenuated total reflection (ATR) sampling assembly. Before the sample analysis, a background spectra was performed with blank ATR crystal. In total, 32 scans were used to collect the spectra in the range from 600 cm^−1^ to 4000 cm^−1^ with a spectral resolution of 4 cm^−1^. Pure PVP powder was also measured as a control for the comparison. Similar parameters were used to monitor the chemical changes associated with stressed UHP-VOR samples.

### 3.7. Thermal Analysis

A simultaneous differential scanning calorimetry-thermogravimetric analysis (DSC-TGA) was performed to determine the moisture content of the PHP complex. Approximately, 10–15 mg solid powder was placed in an aluminum crucible and subjected to thermal analysis. The ramp rate of 10 °C/min was used in the temperature region from 25 °C to 500 °C. Helium was used as a carrier gas with a flow rate of 50 mL min^−1^. The mass changes up to 110 °C in the TGA plot was used to estimate the moisture content. The samples were analyzed in triplicate.

### 3.8. Preparation of Solid Tablet Compacts and Exposure to Accelerated Storage

An equal amount of PHP complex and VOR powder were weighted accurately (50 mg each) in a glass vial and mixed together. The powder mixture (VOR-PHP) was compressed (using compression force of 50 kN for 30 s) into a compact disc using an electrohydraulic hand press (PerkinElmer, Waltham, MA, USA). The compacts were exposed to 40 °C/75% RH in controlled stability cabinets (WTC Binder, Tuttlingen, Germany) up to 10 d. Two separate sets of samples in the closed (with lid) and open (without lid) state were used in glass vials. For each time interval and storage condition, three replicates were used. The sample comprising of as is VOR and as is PVP for each condition was also used as a control set for storage and testing. The BET based surface area was estimated for the compacts.

### 3.9. Analysis of the Stored Samples (VOR-PHP Tablet Compacts)

After each time interval of 1 d, 5 d and 10 d of stress exposure, the samples were withdrawn from the humidity chamber and analyzed using LC for VOR degradation. Each compact was disintegrated and dissolved in 100 mL volumetric flask. A mixture of ACN: water (50:50 *v*/*v*) was used as a diluent. The dissolved API solution was then analyzed by LC.

## 4. Results and Discussion

The VOR was used as a model compound as the susceptibility of VOR to hydrogen peroxide is well documented in a solid state. More precisely, methyl group attached to phenyl ring (para position to sulfur atom) is a site for hydroxylation forming major oxidative degradation products. The chemical purity of the received VOR was found to be >99.9%. The particle size of the received grade of API powder was 372.41 µm (d 50).

### 4.1. LC Analysis

**Figure 1:**
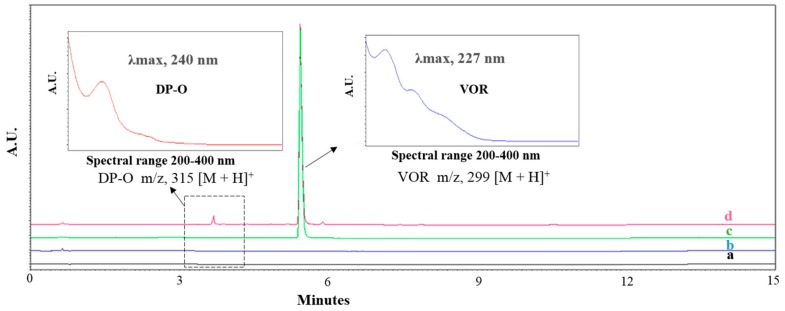
Representative overlay of chromatograms (**a**) Blank sample diluent; (**b**) Blank PVP hydrogen peroxide (PHP) complex; (**c**) vortioxetine hydrobromide (VOR) reference solution; (**d**) VOR-PHP storage sample, showing VOR and oxidative degradation product DP-O (insets denotes UV spectra of drug, VOR and its degradation product, DP-O).

The LC analysis showed a clear separation of the peaks of the drug and oxidative degradation product. The major degradate seen at the relative retention time (RRT) 0.67 was denoted as the oxidative degradation product (DP-O) (Figure 1). The exposed samples at 40 °C/75% RH for VOR-PHP solid compacts showed the presence of DP-O, while it was absent in the control samples (PHP complex, PVP-VOR tablet compacts containing as is VOR and as is PVP). The observations demonstrate the peroxidation of VOR.

### 4.2. Speciation of the Oxidative Degradation Product via Forced Degradation Studies

The oxidative forced degradation studies were conducted to identify and confirm the route and type of degradation reaction in a liquid state (using 30% H_2_O_2_) and in a solid state (using VOR-UHP solid compacts). In both cases, a selective oxidation formed DP-O as the major degradation product. The LC-MS studies for both stressed samples confirmed the formation of hydroxylated product, DP-O. The [M + H]^+^ corresponding to *m*/*z* of VOR and DP-O were 299 and 315 (addition of 16 Da), respectively (MS spectra not shown). The observations were in agreement with the reported case of similar oxidative degradation using MS/MS studies of VOR. As seen in the UV spectra (Figure 1 insets), the addition of hydroxyl group in the DP-O led to a bathochromic shift of the λ_max_ to 240 nm from 227 nm of VOR.

**Figure 2:**
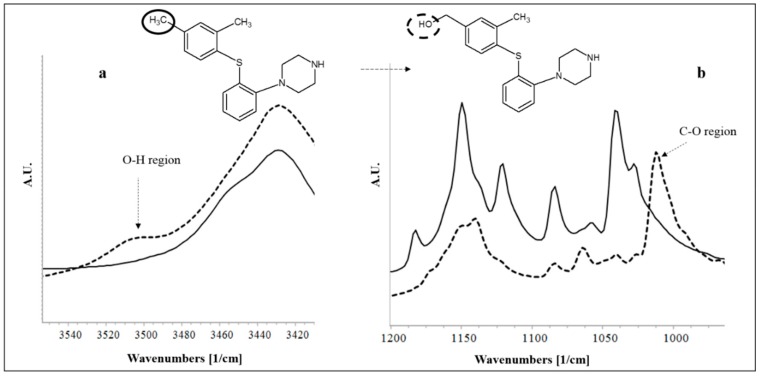
Overlays of partial IR spectra for (**a**) –O–H, (**b**) –C–O vibrational bonds. The dark line represents the unexposed UHP-VOR tablet sample (initial) and the dotted lines represents the UHP-VOR tablet compacts after open exposure to 40 °C/75% RH after 1 day (FD study).

The relative percentage of degradation (DP-O) observed after 1 day of storage at 40 °C/75% RH was enormous in a closed state (42.66 ± 2.67) as well as in an open state (80.03 ± 3.37) storage. Due to the prominent fraction of the degradation product, the functional group signature of the same was noticeable in the infrared spectrum (Figure 2). A distinct vibrational peak appeared in the O-H region (3510 cm^−1^). This is attributable to the addition of hydroxyl to the parent structure (Figure 2a). Similarly, a specific peak was observed at 1013 cm^−1^, corresponding to a C–O vibrational band. These two vibrational bands were absent in the unexposed VOR-UHP compact. This evidenced the addition of an O–H region to the original structure of VOR. The experimental observations suggest that the route and type of degradation product was oxidative (DP-O). The nonreactivity of VOR towards radical induced degradation (we studied using solid AIBN as a stressor) was verified during the feasibility exercise. This proves the selectivity of VOR for peroxide based (solid and solution state) degradation, exclusively. Furthermore, other benefits of PHP over UHP are listed in Table 2.

### 4.3. Physical Characterization and Reproducibility Study of PHP Based Degradation

**Figure 3:**
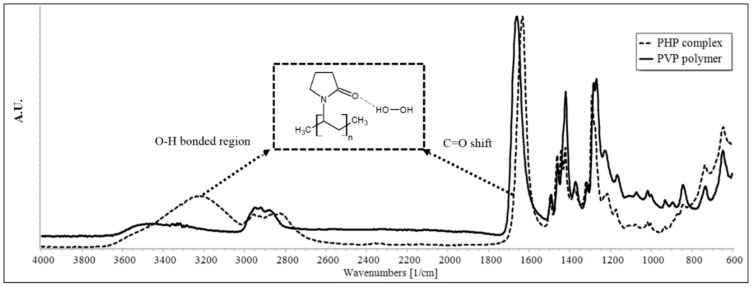
Overlays of the IR spectra of PVP (dark line) and solid stress reagent, PHP complex (dotted line) for characteristics –O–H and –C=O bands.

The characteristic vibrational bands of carbonyl and hydroxyl groups of the PHP complex were distinct in the infrared spectrum of the PHP complex from that of PVP alone (Figure 3). The band of carbonyl group shifted to a lower wavenumber (from 1660 cm^−1^ to 1633 cm^−1^), indicating the formation of a hydrogen bond with hydrogen peroxide (red shift). The hydroxyl (O–H) region of PVP showed a broad band with the maximum at 3500 cm^−1^. This is probably originating from residual moisture and from the O–H present at the ends of PVP chains. While in the PHP complex, an additional and distinct peak with maximum at 3100 cm^−1^ is noticeable, pointing towards the formation of an intermolecular hydrogen bonding between hydrogen peroxide and PVP.

The reproducibility of the PHP complex was ensured by preparing separately three times, following the same procedure. Each time, a consistency in the quality of the complex was confirmed by the identical infrared spectral profiles. The pH of the three separately prepared batches of the PHP complex were 6.65, 6.68 and 6.68. The percent moisture content (free and bound water using thermal TGA method) for PHP was found to be 10.14 ± 0.69. The prepared complex was stored each time at 2–8 °C.

**Figure 4:**
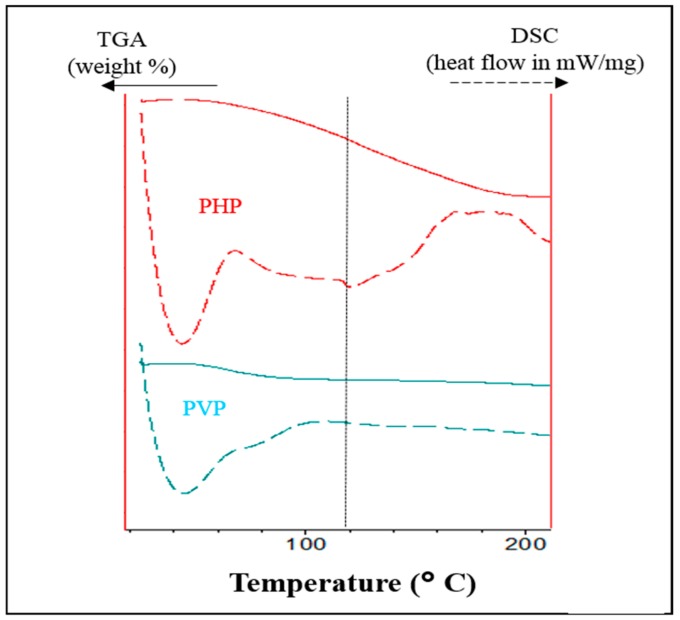
Overlay of thermograms for simultaneous DSC-TGA. The solid lines represent the TGA curve (unit at left Y-axis) and dotted lines with DSC curve (unit at right Y-axis); the curves for PHP marked in the red color line and for PVP in blue color).

The simultaneously recorded DSC and TGA curves in Figure 4 depict the thermal properties of PHP with respect to the pure PVP. The TGA thermograms showed a clear difference in the weight profiles between PHP and PVP alone which is further supported by the DSC desolvation endotherms. For PVP, two endotherms are notable, the first larger one up to 70 °C and the next between 70–100 °C representing the surface moisture and the bound water content, respectively. In the case of PHP, the first sharp peak corresponds the moisture loss followed by a shallow endotherm of a further loss of moisture. Moreover, a small yet notable endotherm is observed at 150 °C. This is possibly a starting temperature of the peroxide loss (boiling point of free hydrogen peroxide is 150 °C).

### 4.4. Outcome of the Solid State Stress Study Using VOR-PHP Study

**Figure 5:**
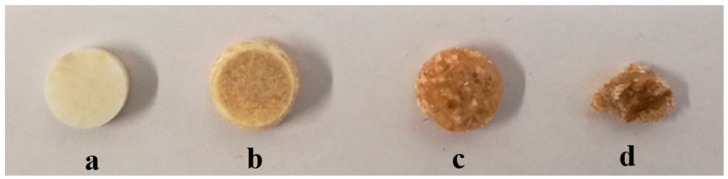
Physical appearance (color) for representative samples of VOR-PHP tablet compacts (**a**) unexposed (initial); and after storage (**b**) 1 d (**c**) 5 d (**d**) 10 d, at 40 °C/75% RH in open state.

The exposed powder compacts in the accelerated storage showed a marked change in physical appearance and the color. The compact changed the color from the initial off-white to brown after stability exposure with the progressive increase in intensity over the storage time. This provided a clear indication of a change in the chemical environment. Beyond 5 d storage, the round shaped compact shrunk to become a sticky mass (Figure 5).

**Figure 6:**
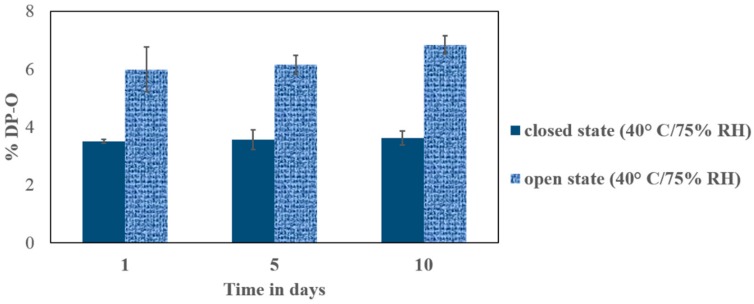
The bar plots representing the percent oxidative degradation in the closed (solid bars) and open (patterned bars) storage at different time intervals. The vertical lines denote the SD for the replicate sample analysis (*n* = 3).

All the PHP treated compacts analyzed using LC indicated the formation of the most prominent oxidative product with RRT 0.67, DP-O (Figure 1). As depicted in Figure 6, the relative percentages of DP-O were 3.51 ± 0.07 (closed)/ 5.99 ± 0.78 (open), 3.56 ± 0.34 (closed)/ 6.15 ± 0.33 (open), and 3.62 ± 0.25 (closed)/ 6.84 ± 0.31 (open) after 1 d, 5 d and, 10 d of exposure, respectively. The PHP mediated peroxidation of VOR was higher upon direct exposure to the moisture, probably due to the moisture sorption by PHP and the release of free H_2_O_2_. However, the extent of degradation was more than halve of the open samples even in the closed sample, where the initial moisture present in the PHP contributed to the reaction. These findings were also in agreement with the observations in the UHP based solid forced degradation (FD) study, where the extent of degradation was almost double than the compared closed state. Regarding the time period of exposure, the extent of degradation after 1 d of exposure was comparable to the later time points, suggesting a stationary state of the solid-state reaction kinetics within 1d. A minor degradation peak (<0.3%) was also noticed at RRT 1.05, which is not characterized in this study. The powder compacts allow the intimate contact between the stress reagent and the drug (BET surface area of ca. 0.92 m^2^/g) to enhance the degradation and minimize the effect of the micromeritic properties as compared to the loose powder blend. The compacts also, to some extent, represent the tablet dosage form where the degradation prediction intended. Furthermore, the different sieved fractions of the powder PHP can be implemented to study the effect of particle contact and surface area on the drug degradation during screening of reactionin a physical blend mixture. In all cases, it is important to normalize the results with an experimentally obtained surface area.

### 4.5. Future Perspective and Practical Relevance of the Stressor

The solid stressor holds the relevance in terms of predicting the chemical instability screening method during solid oral dosage development, since the stress factors, formulation microenvironment and process factors of the final product can be generated to some extent. For example, the stress factors, like the amount of reactive peroxide, RH environment and process relevant factors, like compaction pressure and dwell time, can be independently controlled. The solid PHP complex is safe to handle compared to the existing liquid peroxides. The solid-state oxidative reactivity for the multicomponent system is complicated due to the involvement of various factors, such as the microenvironment, mobility, oxygen diffusion, crystal habits, plasticization by excipients, solid state disorder and salt forms etc. [13,14,15]. The method is simple and can be applicable for the oxidative degradation mechanism in the solid-state formulations containing similar types of excipients. The other common pharmaceutical polymeric excipients, like different molecular weights PVP, cross-linked PVP, soluplus, polycaprolactam are amenable to the formation of molecular complex with hydrogen peroxide. PVP, being one of the most common excipients used in conventional and enabling formulations (e.g., solid dispersion), is a suitable case for a wider range of application for PHP as a solid stressor. In the future, we will also extend the application to study the effect of PHP using multicomponent formulations (e.g., in presence of bulking agent and varying solid state pH modifiers). The proposed method may be potentially applied to study the effect of the lot-to-lot variation among vinyl pyrrolidone type polymeric excipient grades in the multicomponent products. The differential chemical outcomes of solid states (e.g., disordered states of crystalline drug, amorphous content etc.) and varying effects of drug loadings in drug formulations, the quantitative relationship between the stressor content and percent degradation can also be systematically investigated using the present approach. The authors are currently studying some of the latter factors using PHP and VOR that will be the topic of the following publication. This study presents here generic guidance for the use of PHP stressors during the excipient compatibility and/ or conducting a forced degradation study. It is recommended to start with a 1:1 ratio of the PHP complex and drug (as routinely done during binary mixture excipient compatibility design). The prepared blend, preferably in the form of the compact disc, is subjected to accelerated conditions (temperatures and humidity in open (as stress) and a closed state (as a control)). A time period of a few weeks (considering different time points) are selected to achieve a maximum degradation of up to ten percent (a general requirement of forced degradation study). The time to failure approach can be used (considering typical ICH limit for individual impurity <0.2%) for the predictions and statistical evaluation of degradation. The method is also applicable for evaluating the out of specification (OOS), out of trend (OOT) and associated investigational analysis of solid products.

## 5. Conclusions

The present study describes the use of a PVP-hydrogen peroxide molecular complex (PHP) as a reagent to conduct peroxide based stress experiments for solid pharmaceuticals. The molecular complexation through hydrogen bonding was confirmed via infrared analysis. The controlled oxidative degradation of crystalline VOR was achieved using the PHP complex in a powder compact. The degradation reaction tends to attain the stationary phase within a day and is susceptible to the direct exposure of the moisture. Furthermore, the degradation route was selective and no interference from the stress reagent was noticeable for the analysis. The outcome from the present study evidences the feasibility of using a carefully designed excipient-stressor complex as a reagent for screening the oxidative instability during solid formulations development.

## Figures and Tables

**Table 1:** Commonly used oxidative stressors, their scope and shortcomings.

Oxidative Stressor (s)	Description
2,2-azobisisobutyronitrile (AIBN), 4,4-azobis-4-cyanovaleric acid (ACVA), 2,2′-azobis(2-amidinopropane) dihydrochloride (AAPH)	• Radical induced stressors are not applicable to study the peroxide based degradation mechanisms • Nonselective chemical degradation reactions are expected • Additional safety precautions needed while handling in the laboratory (not environment-friendly)
Liquid hydrogen peroxide (H_2_O_2_)	• Do not mimic the solid state mechanisms and reactivity, directly • Corrosive to handle
Transition metals (Cu^2+^ & Fe^3+^)	• Electron transfer mediated (metal catalyzed) redox reaction
Fenton Reagent (solution comprises of liquid hydrogen peroxide and iron (II) sulfate)	• Hydroxyl radicle mediated reactions • Solution state degradation
Polysorbate 80 and Iron (III) solution	• Radical mediated reactions • Solution state degradation
N-methyl pyrrolidone (NMP) as a co-solvent and oxidant	• Solution state degradation • Non-specific degradation, aids side reactions including hydrolysis

**Table 2:** Benefits of PHP over UHP as a solid-state stressor.

Parameter	PHP	UHP
Material attributes for complexation agent	PVP is one of the most commonly used excipients in the pharmaceutical formulations.	Urea is not a common choice in the pharmaceutical formulations.
Selectivity and extent of oxidative degradation reaction	Pure PVP as a byproduct (formed upon dissociation of peroxide from the complex during reaction) is chemically neutral and non-reactive. Secondary degradation reactions are thus not expected.	Pure urea as a byproduct (formed upon dissociation of peroxide from the complex during reaction) will lead to the non-oxidative side reactions by impacting micro-environmental pH. Higher extent of degradation and secondary degradates are expected with this type of matrix.
Context and application towards screening of reactive impurity mediated excipient incompatibilities	Applicable for the formulation containing vinyl pyrrolidone excipients (by externally spiking and creating oxidative environment), studying lot-to-lot variations for different excipient grades. Effect of plasticization, polymer chain length effect on chemical reactivity is feasible in this type of matrix. The study of the mobility and solubility of peroxide reactive impurity in the polymer matrix is feasible.	Not very relevant in this context.
